# Carcinogenic Risk from Lead and Cadmium Contaminating Cow Milk and Soya Beverage Brands Available in the Portuguese Market

**DOI:** 10.3390/jox14020045

**Published:** 2024-06-13

**Authors:** Vanda Lopes de Andrade, Iolanda Ribeiro, Ana Paula Marreilha dos Santos, Michael Aschner, Maria Luisa Mateus

**Affiliations:** 1 Research Institute for Medicines (iMed.ULisboa), Faculty of Pharmacy, Universidade de Lisboa, 1649-003 Lisboa, Portugal; vanda.andrade@esa.ipsantarem.pt (V.L.d.A.); apsantos@ff.ulisboa.pt (A.P.M.d.S.); 2 CERNAS—Cernas—Research Centre for Natural Resources, Environment and Society, Escola Superiora Agrária de Coimbra Bencanta, 3045-601 Coimbra, Portugal; 3 Life Quality Research Centre (CIEQV), IPSantarem/IPLeiria, 2040-413 Rio Maior, Portugal; 4 Faculty of Sciences, Universidade de Lisboa, Campo Grande, 1749-016 Lisboa, Portugal; 5 Department of Molecular Pharmacology, Albert Einstein College of Medicine, Forchheimer Building, Room 209, Bronx, NY 10461, USA; michael.aschner@einsteinmed.edu

**Keywords:** lead–cadmium mixture, cow milk, soya beverages, carcinogenic risk assessment, high consumers, children and adolescents

## Abstract

Our previous work demonstrated the presence of lead (Pb) and cadmium (Cd) contamination in cow milk (CM) and soy beverages (SBs) in Portugal. These metals share carcinogenic mechanisms, suggesting at least additive effects. Our goals were to assess carcinogenic risks from Pb and Cd intake detected in various CM and SB brands on the Portuguese market and to determine the relative contributions of Pb and Cd. Furthermore, we modeled different consumption scenarios for various age/body weight groups to estimate cumulative Excess Lifetime Carcinogenic Risk (ELCR). ELCR was computed by multiplying chronic daily intake by a cancer slope factor for each metal, with an ELCR > 1 × 10^−4^ indicating carcinogenic risk. Five CM and three SB brands posed cancer risks in children, with the highest values at 1.75 × 10^−4^ and 9.12 × 10^−5^, respectively; Pb had mean relative contributions of 87.8 ± 3.1% in CM and 54.9 ± 12.1% in SB. Carcinogenic risks were observed for children, adolescents, and adults in several CM or SB consumption scenarios, albeit at levels above typical Portuguese intakes. Strict monitoring of metal levels, such as Pb and Cd, is advised because CM is a component of many foods, including baby food.

## 1. Introduction

Cow milk (CM) has been widely consumed around the world for thousands of years. Nowadays, its daily consumption is estimated at billions of liters worldwide [1,2]. CM intake may occur across a lifetime in industrialized countries. It represents a habitual intake in many children and adolescents and is frequently encouraged in food-based dietary guidelines, given its balanced nutritional profile [3]. CM consumption can start during pregnancy and continue in infancy, childhood, adolescence, and even in adulthood [4,5]. More recently, due to several issues like lactose intolerance and milk protein allergies arising from CM consumption, an increasing trend for diets that prioritize environmental and health concerns (veganism, flexitarian diet, Paleo, and many others) has increased the demand for plant-based alternatives, as have cultural reasons. The food industry has been addressing such needs by introducing various beverages, which include soy beverages (SBs). Currently, SBs are widely consumed as an alternative to CM and, therefore, a vast number of brands have appeared on the market [2].

However, regions such as the ones under the extensive influence of metal mining and processing industries may have metal contamination in the environment and, subsequently, the food chains [6]. A systematic review from Boudebbouz et al. (2021) [7] noted the presence of metals in CM collected from different regions across the word. It has also been reported that metal accumulation in soybeans is higher than in other beans, which is a cause for health concern [8].

In Portugal, pyrite extraction in mines has a long tradition and represents an important industry which has given rise to several pollution problems, namely, a large volume of mine waste occurring in regions of Portugal as a result of ore extracted from metalliferous mining works during the last 100 years; moreover, abandoned mines are one of the most serious environmental issues occurring in Portugal. Results from soil analysis in Portuguese regions, such as near the Aljustrel mines, show severe soil contamination with metals which include Pb and Cd [9]). Consequent food chain contamination is demonstrated in a Portuguese study using hedgehogs, which as well as other insectivores and small mammals have been used in different European countries to assess heavy metal(loid) pollution. In fact, as their habitats include urban, suburban, rural and natural areas, they are in contact with several sources of metal contamination of soil and water due to industrial, agricultural, and mining activities, among others. In this study, samples of hedgehogs presented alarming Cd levels [10]. Other studies conducted in two Portuguese islands led the authors to conclude the need to set up monitoring and surveillance programs for the safety of cereals and their derivatives in Madeira and the Azores in terms of Pb and Cd [11].

It is well known that Pb and Cd are both toxic for humans, and it is effectively known that the consumption of polluted food is the main source of Pb and Cd intake in the non-smoking population [12,13]. A previous paper from our group addressed how Pb and Cd are metals contaminating both CM and SBs in different areas around the globe, including Portugal. Specifically, we showed metal contamination in several brands of CM and SBs available in the Portuguese market [14]. The absorption of Pb or Cd assures interactions with biological macromolecules, which can lead to adverse health outcomes [15].

Lead (Pb) is one of the major toxic metals in the environment and exposure to it has been associated with the induction of cancer [16,17]. The U.S. Department of Health and Human Services (HHS) has determined that Pb and Pb compounds are reasonably anticipated to be human carcinogens; in addition, the U.S. Environmental Protection Agency (EPA) has classified Pb as a probable human carcinogen and the International Agency for Research on Cancer (IARC) determined that inorganic Pb is probably carcinogenic to humans [18]. Relationships between brain tumors and occupational exposure to Pb have been reported in several countries, including the USA, Finland, Sweden, Australia, and Russia [19]. Studies in Finland and the United Kingdom confirmed a positive association between blood Pb concentrations and several cancers, namely in the brain and lungs [16,19]. 

Cadmium (Cd) is a proven human carcinogen, being, therefore, categorized in group I by the IARC [20] Occupational or environmental Cd exposure has been related to lung, breast, prostate, pancreas, urinary bladder, and nasopharynx cancers. Several other reports have suggested that Cd may also be involved with malignancies of the liver, hematopoietic system stomach cancer, and breast cancer [21,22,23,24,25]. 

Numerous mechanisms have been hypothesized for Pb-induced carcinogenicity, such as DNA damage (single- and double-strand breaks), sister chromatid exchanges, chromosomal abnormalities, micronuclei formation, and cytogenetic disruption [15,26]. Additionally, various mechanisms have been implicated for Cd-induced cancers, and among the most significant ones are the attenuation of apoptosis, inhibition of DNA repair, and alterations in gene expression. Cd may also exert carcinogenic effects by endocrine disruption, cell proliferation, and aberrant DNA methylation [27]. Noteworthy is the fact that both metals share the ability to induce oxidative stress, as through their interactions with living systems, they can produce free radicals which have the potential to harm cellular and molecular structures. Indeed, the basis of carcinogenesis lies in the escalated and sustained generation of reactive oxygen species, leading to chronic oxidative stress [15]. Studies have reported that Pb forms reactive species such as superoxide and hydroxyl radicals as well as hydrogen peroxide, while Cd indirectly generates a multitude of radicals including nitric oxides and, in the same way as Pb, superoxide and hydroxyl radicals and hydrogen peroxide [15,28,29]. Such shared mechanisms by Pb and Cd imply at least an additive effect in cancer induction upon co-exposure scenarios, although a synergistic effect should not be discounted. 

Human health risk assessments have continued to evolve and now focus on the need for cumulative risk assessment, which involves assessing the combined risk from co-exposure to multiple chemicals (and nonchemical stressors). They are broader in scope than traditional chemical risk assessments because they allow for a more comprehensive evaluation of the interaction between different stressors and their combined impact on human health [30]. Nowadays, cumulative cancer risk assessment has become a standard approach, such as for air quality evaluations conducted by the US EPA; in this agency’s framework for air toxics assessment, cancer risks for individual contaminants are treated additively, and cumulative risk is estimated by the mathematical addition of single contaminant risks [31]. Similar approaches have been used by authors such as Badeenezhad et al., 2023 [32] and Radfard et al., 2023 [33], regarding co-exposures to metals through drinking water, by Qing et al., 2023 [34], with focus on co-exposures to metals through foods, and by Abedi et al. (2020) [35], for Pb and Cd concentrations in CM in Iran. 

To our knowledge, no cancer risk assessment due to Pb and Cd contamination of CM or SB in brands available in the Portuguese market has been performed so far, and more particularly considering co-exposure to these two metals. Such assessment is valuable and should be considered from two perspectives: the first one considering the age group, since children (including adolescents) and adults have differences regarding their susceptibility to toxic agents; the second perspective considering the consumer profile, since individuals consuming CM or SB have a metal intake higher than the estimated averages. Although reflective of a smaller population in number, these individuals must also be taken into consideration to protect their health.

Accordingly, the objectives of this work were to assess the carcinogenic risks in three age groups (children, adolescents, and adults) arising from Pb and Cd contamination (i) of different CM and SB brands available in the Portuguese market, and evaluate each metal’s relative contribution; (ii) upon different scenarios of CM or SB daily consumption per age/body weight.

## 2. Methods

### 2.1. Data Treatment

All data were treated using Excel software from Microsoft 365.

### 2.2. Carcinogenic Risk Assessments

#### 2.2.1. Excess Lifetime Carcinogenic Risk

The Excess Lifetime Carcinogenic Risk (ELCR) is the additional risk that someone might have of cancer induction, being also defined as a plausible upper bound estimate of the probability that a person may develop cancer at some point in his or her lifetime following exposure to a contaminant [36].

Following Chen et al.’s 2006 [27] and Badeenezhad et al.’s 2023 [32] methodologies, we assumed an additive carcinogenic effect from Pb and Cd co-exposure. Cancer risk assessment was performed calculating the sum of the ELCR of each metal, according to formula adapted from Badeenezhad et al., 2023 [32] (Formula (1)).

Cumulative ELCR = ELCR _Pb_ + ELCR _Cd_ (1)

Each ELCR was determined according to Formula (2)

ELCR = CDI × CSF(2)

where CDI is the chronic daily intake and the CSF is the cancer slope factor of each metal, with CDI being calculated by Formula (3)

CDI = (C × IR × EF × ED)/(BW × AT)(3)

where C is the concentration of the metal in the food, IR is the ingestion rate, EF is the exposure frequency, ED is the exposure duration, BW is the body weight, and AT is the average time for cancer risk assessment, expressed as ED × 365. Input values used in calculations, and their units, are presented in Table 1. 

The obtained ELCRs were compared with the US EPA recommendations for the health risk level of carcinogenic chemicals: an ELCR value greater than 1 × 10^−4^ indicates carcinogenic risk, a value between 10^−6^ and 10^−4^ corresponds to an acceptable risk interval, and a value below 10^−6^ indicates that the risk is negligible [34].

#### 2.2.2. Metal Levels in Cow Milk and Soya Beverages 

A previous paper from our group showed Pb and Cd contamination in several brands of CM and SBs available in the Portuguese market. Information about the samples analyzed, a detailed explanation of the method employed for sample preparation, the complete analytical procedure for the determination of Cd and Pb by Atomic Absorption Spectrophotometry, the results obtained in each sample, and the parameters determined in the method validation are described by Lopes de Andrade et al., 2023 [14].

### 2.3. Carcinogenic Risk Assessment per Brand of Cow Milk or Soya Beverage 

The cumulative ELCR was determined for each CM and SB brand in three groups with different ages, children (3 to 9 years old), adolescents (10 to 19 years old), and adults [41]. In the children group, an average age of 6 years was considered, corresponding to an average weight of 21 kg, according to data obtained from weight-for-age charts of the World Health Organization (WHO) relative to the 50th percentile [39]. The same procedure was applied to the adolescents group, with an average age of 14 years, corresponding to an average weight of 49 kg [39]. In relation to adults, the age of 85 years, which is the life expectancy in Portugal, was considered. For adults, the average weight of a Portuguese man is 79.5 kg and of a Portuguese woman is 65.8 kg; according to a precautionary principle, we opted to consider a reference weight of 65 kg [38,40]. However, as a child is introduced to CM at approximately 12 months of age (but not before), for all the groups we considered that exposure to metals in CM or SB began at 1 year of age [42].

The amount of intake of CM for each age group was obtained from the National Food, Nutrition, and Physical Activity Survey of the Portuguese General Population, IAN-AF 2015–2016 [37], which was 0.267, 0.250, and 0.150 L for children, adolescents, and adults, respectively. Due to absence of data on SB consumption, it was assumed that the amounts would be similar to CM, especially since SB is frequently consumed as an alternative to CM. 

#### Relative Contribution of Pb and Cd to Carcinogenic Risk 

In each age group and for all the brands for which the determined ELCR was higher than 1 × 10^−4^, indicating carcinogenic risk, the relative contribution of Pb and Cd to the results was evaluated. The results were presented as % of contribution with a basis on the ELCR value of each metal and considering the contribution of both metals as 100%.

### 2.4. Carcinogenic Risk Assessments in Several Scenarios of Daily Consumption of Cow Milk or Soya Beverage per Age/Weight

Again, the cumulative ELCRs were determined, but this time considering the mean Pb or Cd levels found in the 14 brands of the analyzed CM and in the 14 brands of SBs. Several possible daily consumption scenarios, ranging from 0.1 to 1.0 L, were considered for all child ages from 3 to 17 years old. The corresponding weight for each age was obtained from the WHO weight-for-age charts relative to the 50th percentile [39]. For adults, the same possible daily intakes were considered for people weighing from 45 to 90 kg, aiming to cover a wide range of weights which would correspond to Underweight, Normal weight, and Overweight, Degrees I and II, and Obese, according to their body mass indexes.

As mentioned, Table 1 gives the input parameters used to calculate the ERCLs in the several approaches.

## 3. Results

### Carcinogenic Risk Assessments per Brand of Cow Milk or Soya Beverage

**Figure 1:**
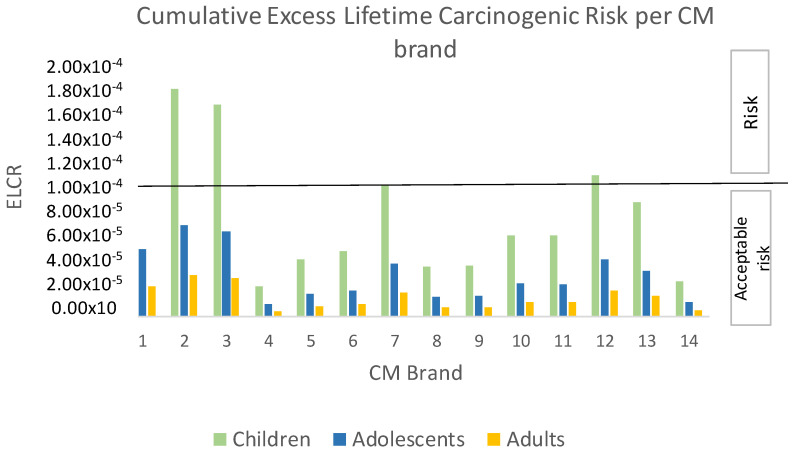
Cumulative Excess Lifetime Carcinogenic Risk (ELCR) due to co-exposure to lead and cadmium contaminating the 14 analyzed cow milk (CM) brands, in children, adolescents, and adults. ELCR values higher than 1 × 10^−4^ imply that there is a risk that a person may develop cancer at some point in their lifetime following exposure to these contaminants.

**Figure 2:**
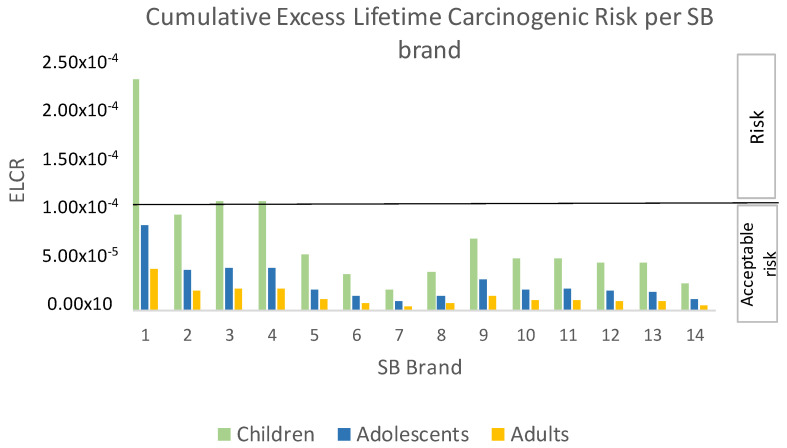
Cumulative Excess Lifetime Carcinogenic Risk (ELCR) due to co-exposure to lead and cadmium contaminating the 14 analyzed soya beverage (SB) brands, in children, adolescents, and adults. ELCR values higher than 1 × 10^−4^ imply that there is a risk that a person may develop cancer at some point their lifetime following exposure to these contaminants.

The consumption of five CM brands, brands 1, 2, 3, 7, and 12, and three SB brands, brands 1, 3, and 4, represented a carcinogenic risk for the children group due to Pb and Cd contamination, when considering the calculated ELCR values higher than 1 × 10^−4^ (Figure 1 and Figure 2). CM brands 1, 2, 3, 7 and 12, exhibited ELCR values of 1.28 × 10^−4^, 1.75 × 10^−4^, 1.63 × 10^−4^, 1 × 10^−4^, and 1.09 × 10^−4^, respectively, while the ELCRs calculated for SB brands 1, 3, and 4 were 2.21 × 10^−4^, 1.04 × 10^−4^, and 1.05 × 10^−4^ (Table 2 and Table 3). Both CM and SB brands for which ELCR values could constitute carcinogenic risk due to Pb and Cd contamination levels had similar values, when considering each mean; CM had a mean ± sd of 1.35 × 10^−4^ ± 3.27 × 10^−5^ and the SB value was 1.30 × 10^−4^ ± 6.10 × 10^−5^.

Owing to the co-presence of Pb and Cd and the assumption of at least an additive effect, the calculated ELCRs were cumulative (Figure 1 and Figure 2). Therefore, we considered it relevant to assess the relative contribution of each metal to the carcinogenic risk of the CM and SB brands which exhibited ELCRs higher than 1 × 10^−4^ for the children group. 

Table 2 shows the relative contribution of Pb and Cd levels in CM for children’s carcinogenic risk, concerning each CM brand with an ELCR value higher than 1 × 10^−4^, and their mean. Pb is the main contributor, since 87.8% ± 3.4% of the cumulative ELCR value is due to Pb levels (Table 2). In a different way, Table 3 shows that the results with respect to SBs are quite heterogeneous, as in brand 1 Pb is the main contributor to a cumulative ELCR value constituting carcinogenic risk (67.2%), but in brands 2 and 3 the relative contribution of Pb and Cd is similar; the ELCR values of Pb are 49.2 and 48.4% of the cumulative (Pb plus Cd) values, respectively (Table 3). 

After considering the carcinogenic risks of each CM or SB brand in the three age groups, children, adolescents and adults, a different approach was adopted. Several possible daily intakes, ranging from low to high consumers (0.1 to 1.0 L/day) in different ages and weights, were considered with regard to the risk of carcinogenicity. 

**Figure 3:**
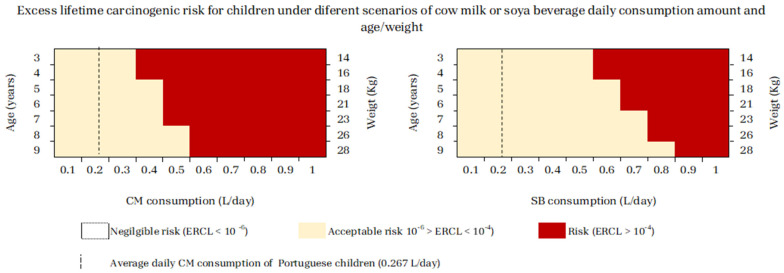
Cumulative Excess Lifetime Carcinogenic Risk (ELCR) for children from 3 to 9 years old, under different scenarios of cow milk (CM) or soy beverage (SB) daily consumption amount per age or weight.

It was estimated that children 3 and 4 years old had an increased risk (ELCR > 1 × 10^−4^) when consuming daily amounts ≥0.4 L/day of CM or ≥0.6 L/day of SBs; the consumption of ≥0.5 L/day of CM or ≥0.7 L/day of SBs presented a risk for children 5 and 6 years old (Figure 3). Additionally, ≥0.5 L/day of CM or ≥0.8 L/day of SBs constituted a carcinogenic risk for 7-year-old children, ≥0.6 L/day of CM or ≥0.8 L of SB for 8-year-old children, and ≥0.6 L of CM or ≥0.9 L of SBs for children 9 years old (Figure 3). All these consumption values were higher than the CM intake estimated for Portuguese children, which was estimated at 0.267 L/day (Figure 3). Such results lead to the consideration that a 3- or 4-year-old child consuming an amount of CM 1.5-fold higher than the estimated 0.267 L/day by the National Food, Nutrition, and Physical Activity Survey of the Portuguese General Population, IAN-AF 2015–2016 [37] might be a risk for carcinogenicity. 

**Figure 4:**
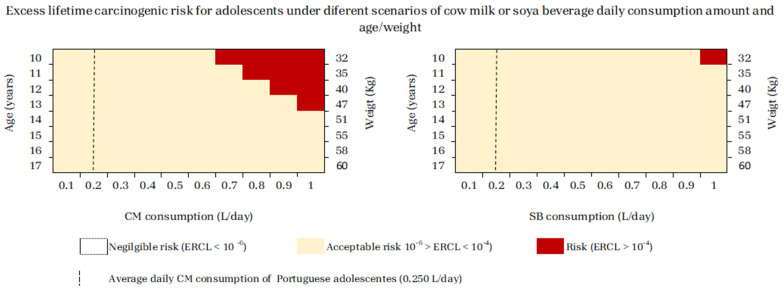
Cumulative Excess Lifetime Carcinogenic Risk (ELCR) for adolescents from 10 to 17 years old, under different scenarios of cow milk (CM) or soy beverage (SB) daily consumption amount per age or weight.

Figure 4 illustrates that adolescents were at risk (ELCR > 1 × 10^−4^) by consuming, daily, ≥0.7 L of CM or 1 L of SBs at 10 years old, ≥0.8 L of CM at 11 years old, ≥0.9 L of CM at 12 years old, and ≥1 L of CM at 13 years old. When considering that the CM intake of adolescents in Portugal is 0.250 L [37], the non-acceptable risk of developing cancer due to Pb and Cd levels found in CM implies a consumption 2.8-fold higher than this value, for a 10-year-old adolescent. 

**Figure 5:**
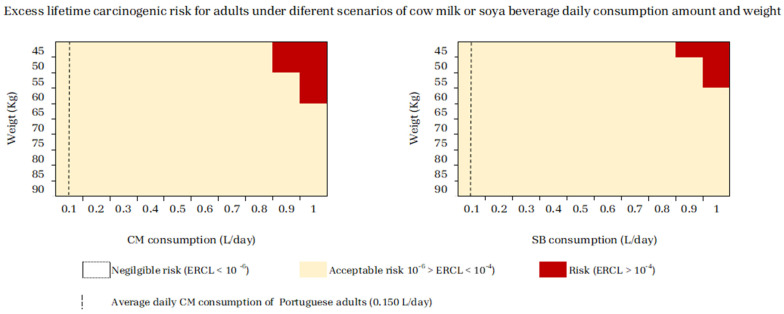
Cumulative Excess Lifetime Carcinogenic Risk (ELCR) for adults under different scenarios of cow milk (CM) or soy beverage (SB) daily consumption amount per weight.

For adults, ELCR values higher than 1 × 10^−4^ were found for persons ranging from 45 to 50 kg consuming ≥ 0.9 L of CM or SBs daily, for persons ranging from 50 to 55 kg consuming ≥0.9 L/day of CM or ≥1 L/day of SBs, and for persons ranging from 60 to 65 kg when consuming ≥1 L of CM daily (Figure 5). Since the CM consumption of Portuguese adults was estimated as 0.150 L/day [37], it can be assumed that drinking 6-fold more CM than this value constitutes a carcinogenic risk for an adult weighing 45 to 50 kg. 

## 4. Discussion

CM is a food included daily in the diet of several million people worldwide because it provides important macro- and micronutrients; it is recognized as an optimal nutrient during childhood and adolescence because of its balanced nutritional value [43]. Nonetheless, CM intake has sharply declined in the last few decades, particularly in developed countries, including most European countries. As an illustration of such a trend, the consumption of CM and dairy products has been decreasing in a progressive fashion, from 56.4 L per capita in 2009 to 50.2 L in 2014 [44]. Concomitantly, the global market for plant-based drinks is experiencing rapid growth, in particular for soy-based (SB) ones, which are gaining popularity among persons with lactose intolerance, milk protein allergies, and vegans [45].

Pb and Cd contamination of CM (and dairy products) are of particular concern [46]. Additionally, beans such as soybeans are known to be prone to accumulating metals [8]. After observing in a previous paper by our group that several brands of CM and SBs available in the Portuguese market are contaminated with Pb and Cd [14], and that both metals are carcinogenic, we assessed such risk in three groups of different ages: children, adolescents, and adults.

### 4.1. Children 

When evaluating each of the 14 CM and 14 SB brands analyzed in our laboratory and considering Pb and Cd levels determined in those samples [14], the consumption of five CM brands and three SB brands constituted a carcinogenic risk (ELCR > 1 × 10^−4^) (Table 2 and Table 3). Of note, such risks were found only for the children group (Figure 1 and Figure 2).

After analyzing the relative contribution of each metal to the carcinogenic risk in brands for which the ELCR was higher than 1 × 10^−4^ (Table 2 and Table 3), we noted that for CM, Pb was the main contributor to the carcinogenic risk, whereas for SBs, both Pb and Cd contributed equally to such risk (Table 2 and Table 3). With respect to Pb in CM, it is noteworthy that physiological differences in its absorption via the gastrointestinal tract have been documented between adults and children; in fact, young children absorb approximately 50% of ingested Pb, while adults absorb only 10% [47].

We also simulated several scenarios of CM or SB daily consumption per age/weight and compared the results with the CM intake estimated for Portuguese children, which according to the National Food, Nutrition, and Physical Activity Survey of the Portuguese General Population, IAN-AF 2015–2016, is 0.267 L/day [37]. Scenarios of acceptable risk (1 × 10^−6^ < ERCL < 1 × 10^−4^) were present in all ages, while any scenario constituted a negligible risk (ERCL < 1 × 10^−6^) (Figure 3). Moreover, for consumers who drink more milk than the average consumers described in the survey conducted in Portugal by Lopes et al. (2018) [41], there were scenarios that constituted carcinogenic risk (ERCL > 1 × 10^−4^), showing that milk consumption posed a risk of cancer at lower intakes than soy-based milk. For example, a child aged 3 or 4 years who consumes 1.5 times more milk than 0.267 L/day can be at risk of carcinogenic effects, while a similar scenario would occur for a child consuming 2.25 times more soy-based milk daily than the assumed 0.267 L/day.

In addition, the consumption of dairy products such as yogurt or cheese needs to be considered. Such foods are dairy products with milk as their main ingredient. While we have been able to determine the levels of Pb and Cd in milk and in soy-based milk accurately, the technological processing of dairy products such as yogurt and cheese affects their concentrations. Nonetheless, it can be expected that approximately similar amounts of these metals exist in these foods. A study conducted by Sujka et al. (2019) [48] in Poland showed the presence of Pb and Cd in most of the analyzed dairy products. In kefir, yogurt, buttermilk, and cream, the content of Pb varied from 0.0039 mg/kg to 0.156 mg/kg. In cheese, the concentration of Pb varied from 0.015 mg/kg to 0.34 mg/kg. We found an average value of 0.0193 mg/L of Pb in the 14 analyzed brands of milk, which, when considering the density of milk as fluctuating between 1.025 and 1.035 g/cm^3^ [49] and an average density value of 1.030 g/cm^3^, allows us to assume average Pb values of 0.0199 mg/kg for milk in our analyzed brands. This value is within the range of Pb contamination of dairy products found by Sujka et al. (2019) [48]. 

This study also revealed the presence of Cd in 50% of the samples, varying in concentration from 0.0067 to 0.0058 mg/kg. By the same reasoning, the average values of the samples analyzed in our work is 0.0022 mg/L (0.0023 mg/kg), which is below the range of Cd contamination found by this author [48]. However, we noted that in the case of milk, Pb was the main contributor to the carcinogenic risk, with an average contribution of 87.8% in brands presenting carcinogenic risk (Table 2). Therefore, when considering the Portuguese Survey performed by Lopes et al. (2018) [37], describing that Portuguese children consume 61 g/day of yogurt and other fermented milks and 18 g/day of cheese, a rough approximation can be made. In fact, summing a consumption of milk of 0.275 kg/day (0.267 L/day) with 0.061 kg/day of yogurt and other fermented milks and 0.018 kg/day of cheese, we obtain an intake of 0.354 kg/day of milk and dairy products by children (see Figure 3). The consumption of dairy products by children is only 28.7% more than the consumption of milk (Figure 3). Thus, we wish to emphasize that the determination of Pb and Cd levels in these foods is necessary to provide a safer interpretation. Regarding soy-based milk, there are no available consumption data to analyze the risk of cancer due to other foods containing soy and apply a similar approach.

Furthermore, according to the Portuguese guidelines for children’s nutrition from 1 to 6 years old from the Portuguese Directorate-General for Health, the recommended daily milk intake is 375 to 500 mL/day [50]. When considering the upper value of 500 mL, this consumption pattern clearly represents a carcinogenic risk for children up to 7 years of age due to the Pb and Cd contamination of the Portuguese milk brands analyzed in this work (Figure 3). When assuming similar consumption of soy-based milk, a daily intake of 500 mL does not represent a risk for the children group (Figure 3).

We should also consider that children are particularly vulnerable to chemicals. Children consume greater amounts of milk than adults (per body weight), or possibly soy-based milk when used as a substitute, making them more susceptible to the risk of exposure to toxic metals [35]. Furthermore, many developmental processes inherent to children’s development, such as cellular migration, synaptogenesis, cell pruning, etc., occur for several years postnatally and are absent during adulthood. Children have disproportionately greater exposures than adults to environmental chemicals and, quite importantly, are particularly sensitive to these exposures because they are poorly equipped to metabolize many toxic compounds. Moreover, they are going through the complex, delicate, and easily disrupted stages of development [51,52]. In addition, children are often more likely to suffer adverse health outcomes from exposure to chemicals for the reasons mentioned above. With respect to cancer, an increased incidence has been seen in children, being the second leading cause of death after physical injuries [52,53].

### 4.2. Adolescents and Adults

As previously mentioned, any of the analyzed CM or SB brands constituted a carcinogenic risk for both adolescents and adults (ELCR < 1 × 10^−4^) (Table 2 and Table 3). 

With regards to the assessment of carcinogenic risks under different scenarios of CM or SB consumption per age/weight in adolescents or per weight in adults, in the same way as observed for the children group, CM consumption posed risk of cancer at lower intakes than SBs (Figure 4 and Figure 5). 

Even so, when taking into account the daily CM consumption values estimated by the National Food, Nutrition, and Physical Activity Survey of the Portuguese General Population, IAN-AF 2015–2016 (0.250 L for adolescents and 0.150 L for adults) [37], adolescents and adults are at lower risk than children. The unacceptable risk for developing cancer due to the Pb and Cd levels found in CM implies a consumption 2.8-fold higher for a 10-year-old adolescent and 6-fold higher for an adult weighing 45 to 50 kg (Figure 4 and Figure 5). 

According to the Portuguese Survey mentioned above, the daily consumption of yogurt and other fermented milks in adolescents and adults is 72 g and 63 g, respectively, while the consumption of cheese is 15 g in adolescents and 19 g in adults [37]. This leads to an estimated CM and dairy product consumption of 0.354 kg/day in adolescents and 0.231 kg/day in adults. Again, from a purely approximate viewpoint, it seems that the contribution of dairy products concomitant with CM consumption does not constitute a relevant risk. When referring to SBs, no consumption data are available to analyze the cancer risk due to other foods that contain soy. 

Nevertheless, according to the Portuguese Association of Nutritionists in 2023 [54], the recommended daily CM consumption is 500 to 750 mL/day. When considering all SB consumption scenarios, these recommended values do not pose any risk for both adolescents and adults (Figure 4 and Figure 5). However, for CM, the upper level of 750 mL constitutes a risk for 10-year-old adolescents, due to the levels of Pb and Cd contamination in these food products, thus posing a cancer risk. 

Finally, this work explored cancer risk considering the more common sources of Pb and Cd exposure from CM and SBs. The fact is that conventional practices in cancer risk assessment (and other risk assessments) frequently involve the analysis of population averages to gauge potential vulnerabilities. However, it is essential to recognize the importance of individual variabilities in this process. Consequently, we carried out the analysis considering different consumption profiles, which included high consumers outside the average range, since these individuals should also be considered in risk assessment. 

Overall, the results obtained in this novel work indicate the need for strict monitoring of metal levels in food products such as CM and SBs.

### 4.3. Environmental Issues

Our results suggest that SB consumption may represent a lower risk of exposure to Pb and Cd, particularly for children, when compared with CM. However, from a holistic perspective, we must remember that “Planetary health is human health” [55], and therefore environmental issues should also be discussed in this paper. It is currently recognized that livestock is disproportionately represented in the emissions profile for the food system, contributing 57% of agricultural emissions. Livestock emissions account for 14.5% of anthropogenic greenhouse gas (GHG) emissions, which increased by 51% between 1961 and 2010. Dairy products rank second in terms of food-related GHG emissions, dominated by methane (CH_4_) and nitrous oxide (N_2_O). Reducing CH_4_ emissions is crucial for achieving climate targets by limiting the extent of warming in the coming decades. In addition, dairy production has a significant effect on water quality through eutrophication and acidification, and biological and chemical pollution, with total global livestock excreta in 2003 estimated to contain 94 million tons of nitrogen. Beyond climate change, water use, and pollution, the dairy sector adversely impacts air quality and significantly contributes to antimicrobial resistance, zoonotic pathogens, land use change-related biodiversity loss, and soil degradation [56]. CM has significantly higher environmental impacts than plant-based alternatives across the metrics of land use, greenhouse gas emissions, water use, and eutrophication. Indeed, CM causes around three times as much greenhouse gas emissions, uses around ten times as much land, two-to-twenty times as much freshwater, and creates much higher levels of eutrophication [57].

## 5. Conclusions

This work showed that children are at greater risk of carcinogenic effects due to Pb and Cd contamination levels in CM or SBs than both adolescents and adults. It was also shown that because of these contaminations there might be a carcinogenic risk for children due to the consumption of some brands of CM or SBs available in the Portuguese market, and that Pb was the main contributor to such risk. In general, CM consumption posed risk of cancer at lower intakes than SBs. Moreover, when considering average Pb and Cd contamination, the upper value of CM consumption recommended by Portuguese guidelines represents carcinogenic risk for children and younger adolescents. We also addressed the potential risk for cancer in high consumers of CM and SBs, showing that this smaller group of the population should be considered. In general, the findings from this study emphasize the importance of closely monitoring the concentrations of metals in food items like CM or SBs, foods widely consumed by the population, and especially children.

## Figures and Tables

**Table 1:** Input values for ELCR calculations.

Parameter	Unit	Children	Adolescents	Adults	Source
Concentration (C)	mg/L	-	-	-	[14]
Ingestion rate (IR)	L/day	0.267	0.25	0.15	[37]
Exposure frequency (EF)	days/year	365 ^(1)^	365 ^(1)^	365 ^(1)^	
Age ^(2)^	years	6	14	85	[38]
Exposure duration (ED) ^(2)^	years	5	13	84	[16]
Body weight (BW) ^(2)^	Kg	21	49	65	[39,40]
Average time (AT)	days	ED × 365	ED × 365	ED × 365	[32]
Cancer slope factor (CSF)	mg/kg/day	Pb: 0.28; Cd: 0.38			[34]

(1) Assuming a daily consumption; (2) Used when ELCRs were calculated for brand of CM and SB.

**Table 2:** Relative contribution of lead and cadmium for the risk of cancer in cow milk (CM) brands that presented carcinogenic risk (ELCR > 1 × 10^−4^) for children.

CM Brand	ELCR			Relative Contribution (%)	
	Pb	Cd	Pb Cd Aditivity	Pb	Cd
1	1.08 × 10^−4^	2.03 × 10^−5^	1.28 × 10^−4^	84.2	15.8
2	1.42 × 10^−4^	3.29 × 10^−5^	1.75 × 10^−4^	81.2	18.8
3	1.42 × 10^−4^	2.08 × 10^−5^	1.63 × 10^−4^	87.2	12.8
7	8.94 × 10^−5^	1.11 × 10^−5^	1.00 × 10^−4^	88.9	11.1
12	1.06 × 10^−4^	2.90 × 10^−6^	1.09 × 10^−4^	97.3	2.7
mean				87.8	12.2
sd				3.4	

**Table 3:** Relative contribution of lead and cadmium for the risk of cancer in soya beverage (SB) brands that presented carcinogenic risk (ELCR > 1 × 10^−4^) for children.

SB Brand	ELCR			Relative Contribution (%)	
	Pb	Cd	Pb Cd Aditivity	Pb	Cd
1	1.48 × 10^−4^	7.23 × 10^−5^	2.21 × 10^−4^	67.2	32.8
3	5.13 × 10^−5^	5.31 × 10^−5^	1.04 × 10^−4^	49.2	50.8
4	5.07 × 10^−5^	5.40 × 10^−5^	1.05 × 10^−4^	48.4	51.6
mean				54.9	45.1
sd				12.8	

## Data Availability

No new data were created or analyzed in this study. Data sharing is not applicable to this article.
